# Comparative Genomic Insights Into the Taxonomic Classification, Diversity, and Secondary Metabolic Potentials of *Kitasatospora*, a Genus Closely Related to *Streptomyces*

**DOI:** 10.3389/fmicb.2021.683814

**Published:** 2021-06-14

**Authors:** Yisong Li, Meng Wang, Zhong-Zhi Sun, Bin-Bin Xie

**Affiliations:** State Key Laboratory of Microbial Technology, Institute of Microbial Technology, Shandong University, Qingdao, China

**Keywords:** *Kitasatospora*, comparative genomics, phylogenomic, inter-genus divergence, diversity, secondary metabolites, recombination

## Abstract

While the genus *Streptomyces* (family *Streptomycetaceae*) has been studied as a model for bacterial secondary metabolism and genetics, its close relatives have been less studied. The genus *Kitasatospora* is the second largest genus in the family *Streptomycetaceae*. However, its taxonomic position within the family remains under debate and the secondary metabolic potential remains largely unclear. Here, we performed systematic comparative genomic and phylogenomic analyses of *Kitasatospora.* Firstly, the three genera within the family *Streptomycetaceae* (*Kitasatospora, Streptomyces*, and *Streptacidiphilus*) showed common genomic features, including high G + C contents, high secondary metabolic potentials, and high recombination frequencies. Secondly, phylogenomic and comparative genomic analyses revealed phylogenetic distinctions and genome content differences among these three genera, supporting *Kitasatospora* as a separate genus within the family. Lastly, the pan-genome analysis revealed extensive genetic diversity within the genus *Kitasatospora*, while functional annotation and genome content comparison suggested genomic differentiation among lineages. This study provided new insights into genomic characteristics of the genus *Kitasatospora*, and also uncovered its previously underestimated and complex secondary metabolism.

## Introduction

The genus *Kitasatospora* belongs to the family *Streptomycetaceae*, a high GC multicellular actinobacterial taxon with a complex mycelial life cycle (Goodfellow et al., 2012; Takahashi, 2017). *Kitasatospora* is one of the three major recognized genera within the family, with the other two genera being *Streptomyces*, which prefers neutral to alkaline soils as a natural habitat (Kämpfer, 2012), and *Streptacidiphilus*, representing a group of acidophilic actinobacteria isolated from acidic soils (Kim et al., 2003). The morphological characteristic of these three genera is rather similar, while *Kitasatospora* is clearly different from the other two in the matter of its cell wall composition as it contains LL- and meso-diaminopimelic acid (DAP), glycine and galactose (Ōmura et al., 1982; Takahashi, 2017). Specifically, the taxonomy status of the genus *Kitasatospora* has been considered controversial for many years (Ōmura et al., 1982; Wellington et al., 1992; Zhang et al., 1997). Recently, more detailed genetic analyses based on morphology-related genes, including *bldB*, *mbl*, *whiJ*, and *murE*, and also house-keeping genes provided evidence that it should be regarded as a separate genus (Girard et al., 2014; Labeda et al., 2017). To date, more than 33 species in the genus *Kitasatospora* are validly published, many of which have been identified in recent years (Parte, 2018).

Members of *Streptomycetaceae* are well known as rich sources of multifarious secondary metabolites, the majority of which are derived from the genus *Streptomyces* (Hopwood, 2007; Barka et al., 2016). Meanwhile, *Streptomyces* could inhabit various habitats, providing ideal materials for studies of microbial ecology and evolution. Therefore, *Streptomyces* species have received enormous attention in microbial sampling efforts. It has also been shown that *Streptomyces* genomes are recombinogenic, with a high recombination rate exceeding those observed within many other bacterial species (Doroghazi and Buckley, 2010). As the sister genus of *Streptomyces*, *Kitasatospora* has gradually attracted people’s attention (Hao et al., 2020; Kim et al., 2020; Yun et al., 2020). So far, at least 50 bioactive compounds have been discovered from *Kitasatospora* strains (Takahashi, 2017), such as setamycin, kitasetaline, and satosporin (Ōmura et al., 1981; Aroonsri et al., 2012; Arens et al., 2013), exhibiting a wide range of bioactivities (e.g., antifungal, herbicidal, antitumor). Nevertheless, little is known about the potential differences in the secondary metabolism and genetics between genera.

This study was aimed (1) to investigate the phylogenetic and taxonomic position of *Kitasatospora*, and (2) to explore the secondary metabolic potentials of *Kitasatospora*. To address the former issue, phylogenomic analyses were performed and genome content were compared between *Kitasatospora* and the closely related genera. To address the later, secondary metabolite biosynthetic gene clusters were annotated and compared in *Kitasatospora* genomes. This study represented the first comprehensive comparative genomic study of the genus *Kitasatospora*.

## Materials and Methods

### Genome Data Set

All genomes of the members of the family *Streptomycetaceae* (April 2020) were downloaded from the NCBI genomes FTP site^1^. Genomic qualities of those genomes were assessed by using CheckM (Parks et al., 2015), and only genomes that were at least 95% complete and had no more than 5% contamination were screened to the further study. Taxonomy assignment of these genomes was performed using the Genome Taxonomy Database (GTDB) toolkit (Chaumeil et al., 2019). To ensure that each genus contained enough genomes to allow statistically robust analysis, genomes were dereplicated using dRep (Olm et al., 2017), with 99.5% average nucleotide identity (ANI) for *Kitasatospora* and 95% for *Streptomyces* and *Streptacidiphilus*. Through these, a total of 45, 575, and 12 high-quality and non-redundant genomes of strains belonging to *Kitasatospora, Streptomyces*, and *Streptacidiphilus* were retained. The information of isolation source was obtained from the NCBI BioSample database and from the literature. Similarity searches for the 16S rRNA gene sequences were performed by the EzBioCloud server (Yoon et al., 2017). Whole genome ANI for each pair of genomes was computed by FastANI (Jain et al., 2018).

### Genome Annotation and Determination of Homologous Gene Families

To avoid bias due to differences in gene calling and annotation, all assemblies were reannotated using Prokka (Seemann, 2014). To improve the accuracy of genome annotation, function annotation and classification of proteins were performed by sequence comparison using DIAMOND BLASTP (*E*-value 1e-05, coverage 0.5, and identity 50%) (Buchfink et al., 2015) against the recently updated Clusters of Orthologous Group (COG) database (Galperin et al., 2020). If a gene was assigned to more than one COG category, each COG category was calculated separately. PFAM domains of proteins were further identified using PfamScan (El-Gebali et al., 2019) with default parameters. Insertion sequences were identified by BLASTP against the ISFinder database (*E*-value 1e-05) (Siguier et al., 2006). Via the use of GET_HOMOLOGUES (Contreras-Moreira and Vinuesa, 2013) with OrthoMCL (Li et al., 2003) clustering algorithm, homologous gene families were calculated, and cloud, shell, and (soft-) core pangenome components were also derived. Pan-genome statistics were visualized using PanGP (Zhao et al., 2014).

### Phylogenomics and Population Structure Analyses

To build the phylogenetic tree of the family *Streptomycetaceae*, we used the GToTree (Lee, 2019) package with default parameters and the “*Actinobacteria*” HMM-set. Through this, a concatenated protein alignment from the 138 marker genes was constructed using Muscle (Edgar, 2004) and subsequently trimmed using TrimAl (Capella-Gutiérrez et al., 2009). The approximately maximum-likelihood phylogenetic tree was built with FastTree (Price et al., 2010). We also constructed a marker-based phylogenetic tree of the genus *Kitasatospora* by using the GET_PHYLOMARKERS pipelines (Vinuesa et al., 2018) run in default mode, identifying high-quality marker genes for robust phylogenomic analyses based on nucleotide sequences of the genes from the resulting single-copy core genome. Within the pipelines, the final phylogenetic tree was generated by ML algorithms with the best model of evolution GTR + F + ASC + R4 in the IQ-TREE software (Nguyen et al., 2015) based on the concatenated gene sequences with 1,000 bootstrap replicates. The tree topology was visualized using the Interactive Tree of Life (Letunic and Bork, 2016). In addition, we used R function pvclust (Suzuki and Shimodaira, 2006) to perform a hierarchical cluster analysis, based on an absence/presence (0/1) matrix of dispensable genes according to GET_HOMOLOGUES results. K-medoids clustering was performed by using the partitioning around medoids (PAM) algorithm from the R package fpc^2^. Population structure was assessed using hierarchical Bayesian analysis of population structure (BAPS) (Corander et al., 2004).

### Genome Mining for Secondary Metabolite Biosynthetic Gene Clusters

Prediction of secondary metabolite biosynthetic gene clusters (smBGCs) was performed using the antiSMASH 5.0 software (Blin et al., 2019), with annotations based on the MIBiG repository (Medema et al., 2015). The final BGC set was analyzed using BiG-SCAPE (Navarro-Muñoz et al., 2020) under –hybrid and –mode glocal settings at distance cutoffs of 0.3, 0.5, and 0.7. The networks were visualized with Cytoscape (Shannon et al., 2003). We applied COUNT (Csürös, 2010) under the Wagner parsimony algorithm for ancestral reconstruction of secondary metabolite biosynthetic gene cluster families (smBGCFs), and for detecting the gain and loss events that might have occurred during the evolutionary history the genus *Kitasatospora*.

### Estimation of Genetic Recombination

SplitsTree (Huson and Bryant, 2006) was used to construct the network with the NeighborNet algorithm, and also to calculate the pairwise homoplasy index (Bruen et al., 2006). Ancestral and recent recombination events happening in each single-copy core gene were identified using fastGEAR (Mostowy et al., 2017) with default parameters. Recombination parameters, including the fraction of sample diversity derived from recombination (c) and the relative rate of recombination to mutation (γ/μ) were calculated by mcorr (Lin and Kussell, 2019) with default parameters, based on the single-copy core genes. Fifty subsets were constructed, each containing 50 different species (pairwise ANI < 95%; 20 for genus *Kitasatospora*, 20 for genus *Streptomyces*, and 10 for genus *Streptacidiphilus*) randomly selected from the genome dataset.

### Statistical Analysis

Variation in the functional profiles was performed using non-metric multidimensional scaling (NMDS) analysis on Bray-Curtis dissimilarity matrices with the “ordinate” function in the phyloseq R package (McMurdie and Holmes, 2013). The significance of the differences in functional profiles was tested by permutational multivariate analyses of variance (PERMANOVA) using the “adonis” function in the vegan R package^3^ with 999 random permutations. SIMPER analysis was employed with the “simper” function of the vegan R package to determine the most differentiating COG entries among different genome groups. Statistical analyses, including Wilcoxon test, Kruskal-Wallis test, and Student’s *t*-test, were performed in R.

## Results

### Phylogenetic Position of *Kitasatospora*

**FIGURE 1:**
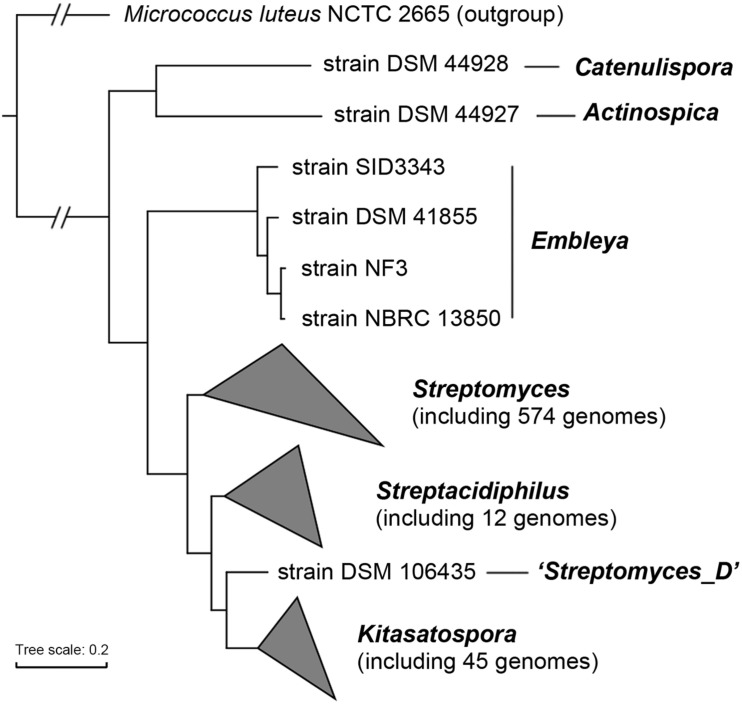
Phylogenomic tree of the family *Streptomycetaceae*. The tree was constructed based on 138 single-copy actinobacterial marker genes. Taxonomy of each strain was derived from GTDB. All nodes have 100% bootstrap support. The scale bar indicates 20% sequence divergence.

Our dataset contained genomes for 45 *Kitasatospora* strains, including five complete and 40 draft genome sequences, and the characteristic genomic features are summarized in Supplementary Table 1. Using 138 single-copy actinobacterial marker genes, we examined the phylogenetic relatedness of the entire family *Streptomycetaceae* (Figure 1). The result showed a good correlation with those observed based on 16S rRNA gene and multi-locus sequences (Labeda et al., 2017; Takahashi, 2017). Generally, strains were clustered based on their source of genus, supporting the current taxonomic status of *Kitasatospora* as a separate genus. The only exception was strain DSM 106435, which has been proposed as the type strain of *Streptacidiphilus bronchialis* (Nouioui et al., 2019) and has been classified as “*Streptomyces_D*” in GTDB. However, phylogenomic analysis indicated that this strain was more closely related to *Kitasatospora*.

### Inter-Genus Comparison

The genome size of *Kitasatospora* ranged from 6.3 (strain NRRL ISP-5024) to 12.3 Mbp (strain CB01881), with a mean size of 8.7 ± 1.0 Mbp, similar to those of the other two genera (Supplementary Figure 1A). Notably, the G + C content varied between 71.2 and 74.5% with an average of 73.1 ± 0.8%, which was significantly larger than those of *Streptomyces* and *Streptacidiphilus* (71.7 ± 0.9% and 71.8 ± 0.7%, respectively; Kruskal-Wallis test, *p* < 0.001; Supplementary Figure 1B). Calculation of ANI revealed higher intra-genus ANI values of *Kitasatospora* (>85.7%) than inter-genus values (<81.7% for *Kitasatospora*-*Streptomyces* and <80.9% for *Kitasatospora*-*Streptacidiphilus*), suggesting boundaries between genera. It was noted strain DSM 106435 showed ANI values of ∼81.1% (coverage, ∼43.5%) with the *Kitasatospora* clade and of ∼80.2% (coverage, ∼46.2%) with the *Streptacidiphilus* clade, all of which were close to the above inter-genus values. This result, together with the unique phylogenetic position of strain DSM 106435, suggested that strain DSM 106435 may represent a new genus.

**FIGURE 2:**
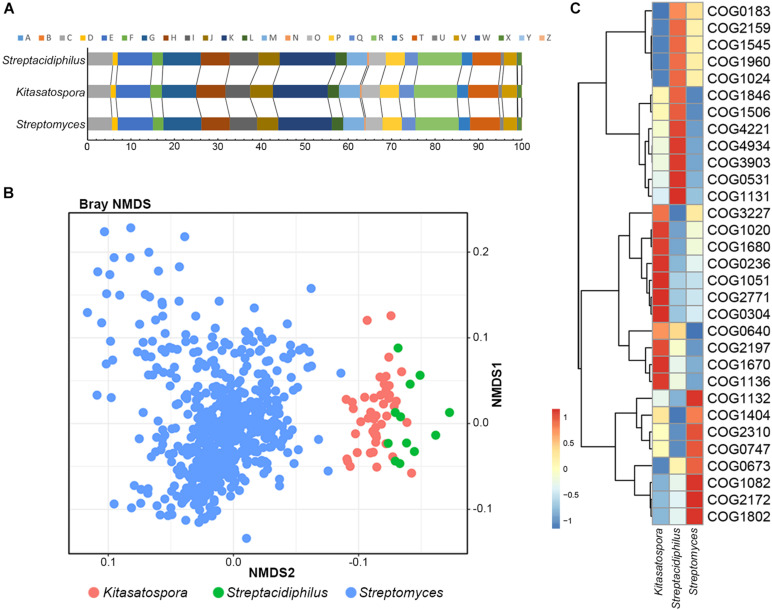
Correlation and divergence of functional traits between genera. **(A)** Stack bar chart showing functional proportions of genes based on COG categories. **(B)** Non-metric multidimensional scaling (NMDS) plot of COGs showing distinct clustering of each genus. **(C)** Heatmap of COGs that significantly contributed most to the dissimilarity between different genera. Relative abundance of the COGs is presented after transformation with a z-score.

Clusters of Orthologous Group annotations of 45 *Kitasatospora* and 587 non-*Kitasatospora* representative genomes were used as input to assess the correlation and divergence of functional traits between genera. Based on the proportions of each COG category, no significant differences were detected between the three genera (PERMANOVA test, the significance level *p* = 0.05; Figure 2A). However, based on the number of proteins annotated to each COG entries, the three genera showed different functional profiles (PERMANOVA test, *p* = 0.001; Figure 2B).

A total of 31 COGs were detected that significantly contributed most to the dissimilarity between genera (SIMPER analysis, >0.05% contribution, *p* < 0.01; Figure 2C and Supplementary Table 2). Particularly, about half (14/31) functional attributes were related to transcription (COG K), lipid transport and metabolism (COG I) and posttranslational modification, protein turnover, or chaperones (COG O). For example, five transcription/response related gene families (COG2771, COG0640, COG1846, COG1802, and COG2197) made great contribution to the separation of *Kitasatospora* and *Streptomyces*: *Kitasatospora* contains more genes of transcriptional regulator families CsgD, ArsR, MarR and NarL/FixJ, while *Streptomyces* contains more GntR, suggesting potential differences in the regulatory mechanisms. For lipid metabolism, two differentiating COG entries (COG0304 and COG0236), implicated in type II fatty acid biosynthesis, were more abundant in *Kitasatospora*, whereas COG1960, COG1024 (both involved in the carnitine metabolism) and COG0183 (participating in the Acetyl-CoA acetyltransferase activity) were more abundant in the other two. There were also four COGs related to posttranslational modification, protein turnover, or chaperone functions, including COG3227 (Zn-dependent metalloprotease), COG1670 (protein N-acetyltransferase), COG4934 (serine protease), and COG1404 (serine protease). Among them, COG4934 has been reported to pertain to acidity linked genes in archaea (Gonzalez and Zimmer, 2008). Consistently, as the most acidophilic genus in *Streptomycetaceae* (Goodfellow et al., 2012), *Streptacidiphilus* has the largest number of copies (10.6, averagely), compared with only 1.6 and 4.7 copies in *Streptomyces* and *Kitasatospora*, respectively. In addition, genes of COG1545, which encoded an uncharacterized OB-fold protein, only existed in 16 (35.56%) *Kitasatospora* strains with only one copy (pairwise amino acid sequence identity >52.10%), but were presented in all strains of *Streptacidiphilus* and 73.39% of strains of *Streptomyces*, with an average of 5.58 (median, 5) and 4.87 (median, 5) copies, respectively, and shared a low identity (amino acid sequence identity <45.20%) with the former.

### Intra-Genus Diversity

**FIGURE 3:**
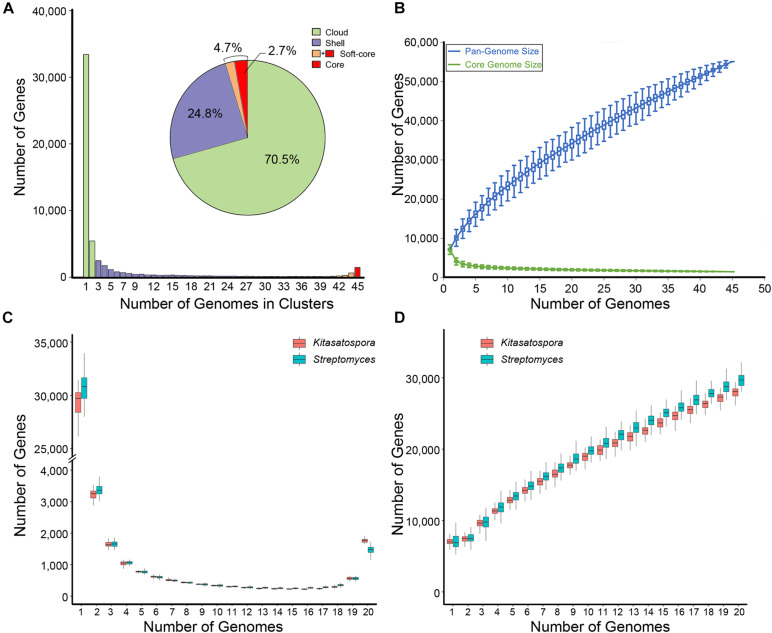
Pan-genome profile of *Kitasatospora*. **(A)** Histogram distributions of cloud, shell, and (soft-) core genes. Pie chart displays percentage of each part of the total genes. **(B)** The sizes of pan- and core-genomes in relation to numbers of genomes added into the gene pool. **(C,D)** Comparison of the pangenome features between genera *Kitasatospora* and *Streptomyces*. The data was calculated from 50 subsets each containing 40 different species (pairwise ANI < 95%; 20 from *Kitasatospora* and 20 from *Streptomyces*) randomly selected from the genome dataset. **(C)** Distribution of genes across strains. **(D)** The sizes of pan-genome in relation to numbers of genomes added into the gene pool.

The number of coding sequences per genome of *Kitasatospora* ranged between 5,482 and 9,863 (mean, 7,568 ± 748). Based on the gene content table obtained by GET_HOMOLOGUES (Contreras-Moreira and Vinuesa, 2013), the genus *Kitasatospora* had a pan-genome of 55,080 gene families (Figures 3A,B). Among these, 1,476 families (2.7% of total) were presented in all strains (core genome), and 2,563 families (4.7%, including the core genome) were conserved in more than 93% of all strains analyzed here (soft-core genome), which represented 26.3 to 47.1% of the gene content of each strain (35.7%, averagely). The cloud genes (rare genes presented only in one or two strains) contained 70.5% of the pan-genome (38,847 families) and accounted for 13.5% for each strain, averagely. The remaining 24.8% were shell genes (13,670), making up about half the genome content for each strain, averagely. Thus, the flexible content (cloud plus shell genes) formed more than 95% of the pan-genome. The pan-genome fitting curve did not reach a plateau as more genomes were sampled (α = 0.61, estimated by power-law regression), suggesting that the pan-genome of *Kitasatospora* was open (Figure 3B). Correspondingly, the pan-genome still increased with more than 700 new genes after addition of a 45th genome. This pan-genome structure was comparable to that of *Streptomyces* (Figures 3C,D). All these results indicated that the gene content of the genus was highly diverged.

**FIGURE 4:**
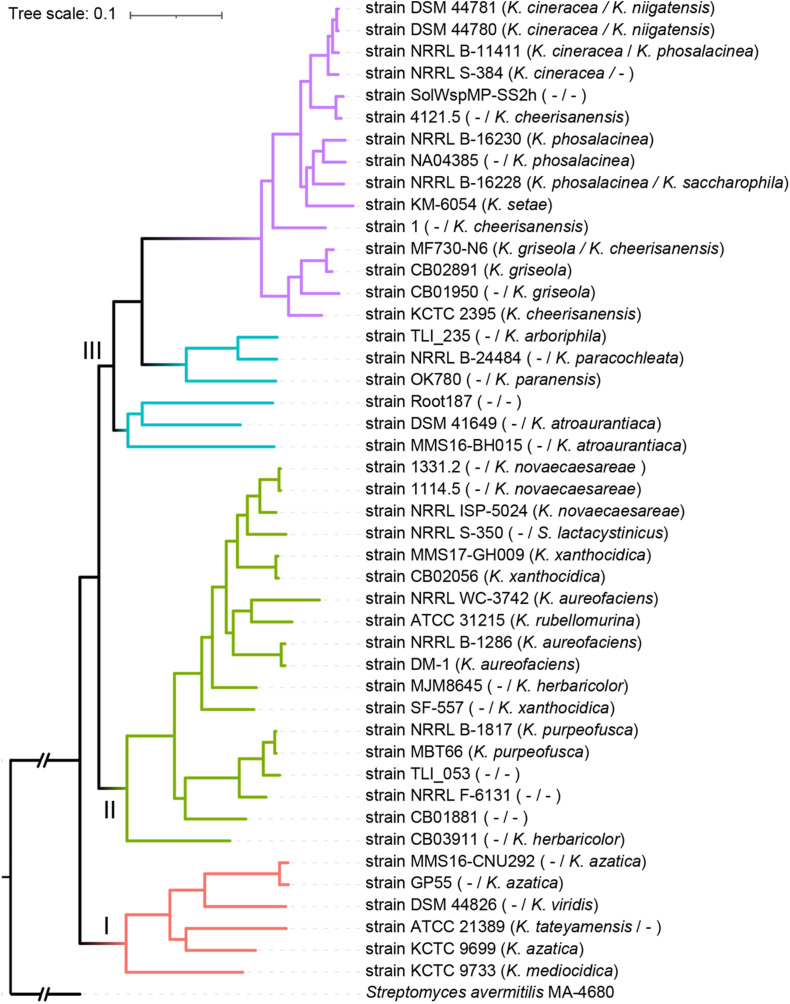
Maximum-likelihood phylogenomic tree of the genus *Kitasatospora*. The tree was constructed based on 456 core genes that could give a well-resolved tree topology and that did not show signs of recombination. Four major lineages were presented by different branch colors (red, Lineage I; green, Lineage II; cyan, Lineage III; purple, Lineage IV). Taxonomy of each strain derived from GTDB and 16S rRNA gene was indicated on left and right of the slash in the bracket, respectively (if consistent, only one was shown). “I,” “II,” or “III” indicates genetic clades. All nodes have 100% bootstrap support. The scale bar indicates 10% sequence divergence.

To further evaluate the intra-genus differentiation of the genus, phylogenomics reconstruction was performed based on 456 high-quality phylogenomic marker (core) genes. As shown in Figure 4, the phylogenomic tree was well-supported, showing three well-defined genetic clades: Clades I, II, and III. A hierarchical clustering tree based on the content of dispensable genes showed three clusters generally corresponding to the three clades in the core gene tree (Supplementary Figure 2), indicating the existence of inter-clade gene content difference within the genus. In the core gene tree, 15 strains formed a monophyletic lineage (Figure 4, purple, named as Lineage IV) that diverged deeply from the other strains (Figure 4, cyan, Lineage III, paraphyletic) in Clade III. Similarly, these 15 strains were clustered into a single branch (Supplementary Figure 2, purple) that was different from the main branch of other strains (Supplementary Figure 2, cyan) from Clade III of the core gene tree. This result was generally consistent with the k-medoids clustering and population structure analyses, both of which showed differences within the Clade III (Supplementary Table 1). Therefore, in addition to the inter-clade differentiation, differentiation between Lineages IV and III (both within Clade III) has merged. The intra-genus difference was further investigated based on the four lineages: Lineages I (corresponding to Clade I), II (corresponding to Clade II), III, and IV.

**FIGURE 5:**
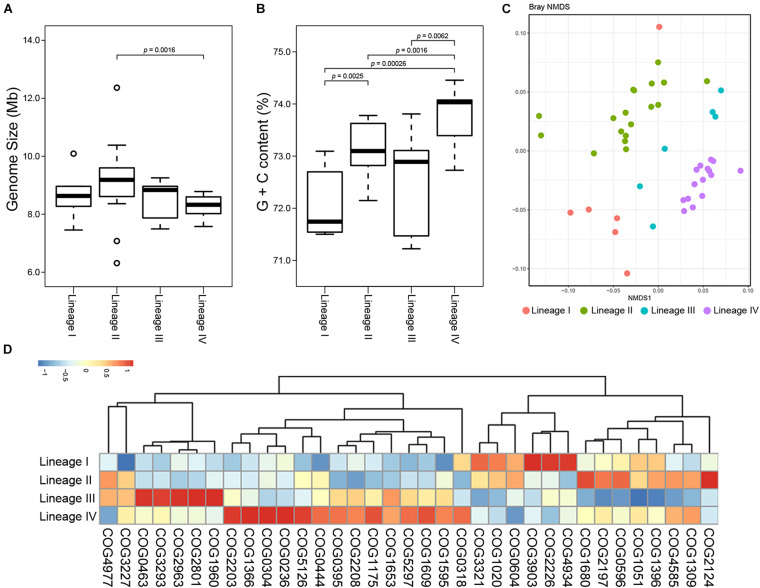
Divergence between lineages of *Kitasatospora*. Variations in genome size **(A)** and G + C content **(B)** between genera in family *Streptomycetaceae*. The indicated *p* values were calculated using Wilcoxon test. Only *p* values less than 0.05 are shown. The boxplot shows the median, and the first and third quartiles as the lower and upper hinges. Outliers are indicated as dots. **(C)** Non-metric multidimensional scaling (NMDS) plots of COGs showing distinct clustering of lineages within *Kitasatospora*. **(D)** Heatmap of COGs that significantly contributed most to the dissimilarity between different lineages within *Kitasatospora*. Relative abundance of the COGs is presented after transformation with a z-score.

We next sought to detect the inter-lineage differences. Genome sizes of the lineages were largely same (Figure 5A). The G + C content of Lineage IV was higher than those of others (Figure 5B). Functional profiles were also significantly different between the four lineages (PERMANOVA, *p* = 0.001; Figure 5C). A total of 35 COGs were detected which greatly promoted the separation between lineages (Figure 5D), ten of which also showed a differential distribution among lineages within *Kitasatospora* (Supplementary Table 3). For example, COG4934, the acidity linked genes mentioned before, showed similar abundance in Lineage I to that in *Streptacidiphilus*. This gene was presented with on average 10.2 copies in Lineage I, while less than 5.2 in Lineages II to IV, suggesting that Lineage I might be more suitable for acidic environments than other lineages. Similarly, a predicted ATPase-coding gene (COG3903) was also more abundant in Lineage I and *Streptacidiphilus* than other lineages.

### Great Potential and Diversity of Secondary Metabolism in *Kitasatospora*

To assess the genomic repertoire for secondary metabolite biosynthetic gene clusters (smBGCs), we performed a computational genome mining analysis using the program antiSMASH (Blin et al., 2019). A total of 1,535 smBGCs were predicted, with 22 – 50 (mean, 34.1 ± 8.2) smBGCs per genome, and the total length of these gene clusters occupied 8.7 – 24.7% (mean, 15.6 ± 3.9%) of the whole genome (Supplementary Figure 3). The number and percentage of length in *Kitasatospora* were significantly larger than those in *Streptomyces* (30.9 ± 7.9; 13.2 ± 4.0%; Wilcoxon test, *p* = 0.027 and *p* = 0.00014, respectively) and *Streptacidiphilus* (27.8 ± 6.8; 10.6 ± 3.2%; *p* = 0.068 and *p* = 5.816e-05, respectively). Difference was also observed between the *Kitasatospora* lineages (Kruskal-Wallis test, *p* = 0.0025): Lineage II contains on average more smBGCs than Lineages III and IV (Wilcoxon test, *p* < 0.05; Supplementary Figure 4). These 1,535 *Kitasatospora* smBGCs could be grouped into 8 major biosynthetic classes (Supplementary Figure 4), with an average of 19.6% RiPPs, 17.2% NRPS, 14.7% terpene, 7.3% PKS-NRP_Hybrids, 6.5% “PKSother”, 4.7% type I PKS, 0.25% saccharide, and 29.6% others (non-classifiable) in each strain. Compared with *Streptomyces*, *Kitasatospora* are significantly richer in classes RiPPs, NRPS, and “PKSother”, but poorer in “Others” (Wilcoxon test, *p* < 0.05).

In order to gain insights into the diversity of smBCGs, similarity networks were constructed using the BiG-SCAPE algorithm (Navarro-Muñoz et al., 2020), which defined “secondary metabolite biosynthetic gene cluster families” (smBGCFs) based on their Pfam similarities. A total of 896, 245, and 9,215 smBGCFs were detected (*c* = 0.3) for *Kitasatospora*, *Streptacidiphilus* and *Streptomyces*, respectively. Comparison of the presence and absence of these smBGCFs revealed that the three genera had highly divergent secondary metabolic profiles (PERMANOVA, *p* = 0.001). For the genus *Kitasatospora*, up to 92.9% of the total smBGCFs were genus-specific, and only 2.3% shown homology to MIBiG entries. Only 2.2% (*n* = 20) were conserved in more than 20% of strains, and more than three-quarters (*n* = 692, 77.2%) were singletons, suggesting a broad secondary metabolic diversity within the genus. Even by raising the default similarity score cutoff from *c* = 0.3 to *c* = 0.7, the proportion of conserved smBGCFs did not change a lot (5.9%), and still more than half (51.4%) were singletons.

To further assess the smBGCFs diversity in *Kitasatospora*, we chose to use a relatively stricter raw distance cutoff of 0.3. We firstly investigated the gain/loss events of smBGCFs that might have occurred during the evolutionary history of the genus *Kitasatospora* (Supplementary Figure 5). The family numbers showed a noticeable upward trend, with 1,043 gains while only 32 losses. No smBGCF was inferred at the last common ancestor of the genus, and no more than three were inferred at the nodes of the three major clades. We also noticed that 81.40% of total gain events and 62.50% of total loss events occurred at terminal nodes. In addition, within each genome, between 14.29 and 42.86% of the smBGCs (mean, 26.92%) comprised one or more genes that were annotated as mobile genetic elements (MGEs) such as insertion sequences, prophages or transposases. These results suggested that the smBGCs of *Kitasatospora* have experienced remarkable expansion, perhaps mediated by HGT, in the evolutionary history.

**FIGURE 6:**
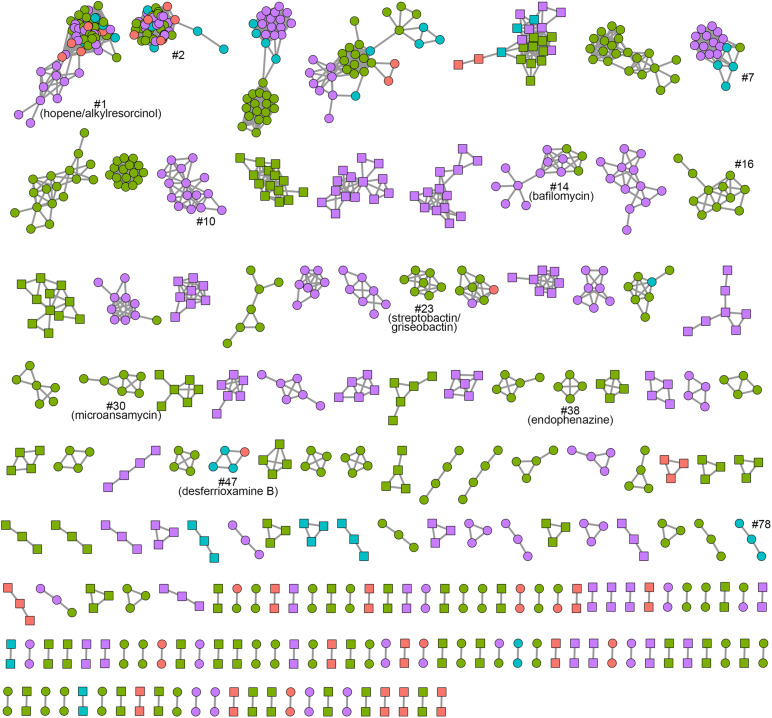
BiG-SCAPE sequence similarity network of *Kitasatospora*. Each node represents one BGC identified by antiSMASH. Colors represent lineages I to IV (red, Lineage I; green, Lineage II; cyan, Lineage III; purple, Lineage IV). *Kitasatospora*-specific BGCs are highlighted with square node. BGCs discussed in this work are marked.

The network of non-singleton smBGCFs was also built (Figure 6 and Supplementary Figure 6). A total of 178 networks were formed. Two networks, networks 1 and 2, contained smBGCs from all the 45 *Kitasatospora* strains. Network 1 included two types of clusters: a hopene biosynthetic gene cluster, which was found in all *Kitasatospora* genomes, and a type III PKS gene cluster, which was mainly distributed in Lineage IV and included three conserved biosynthetic genes from the alkylresorcinol biosynthetic gene cluster. Network 2 summarized pathways that were likely responsible for the formation of an uncharted siderophore. The main genetic structures of these three clusters above were highly homologous to those in *Streptomyces*. Only a small number of networks contained members that showed similarities to reported clusters. For example, smBGCs from Network 7 were predicted to encode unknown linear azole-containing peptides (LAPs), which displayed similarities with those in *Verrucosispora maris* and *Herbidospora* spp.; Networks 10, 16, and 78 contained predicted spore pigment polyketide gene clusters; and Network 47, contained a well-studied desferrioxamine B gene cluster, which was very common in *Streptomyces*. Other well-annotated smBGCs also included members encoding streptobactin/griseobactin (Network 23), bafilomycin (Network 14), microansamycin (Network 30), and endophenazine (Network 38). Furthermore, most of the abundant *Kitasatospora*-specific networks were lineage-specific (Figure 6, networks with nodes in the same color). These results revealed a huge diversity and potential of secondary metabolite production that may be harnessed through dedicated mining of *Kitasatospora.*

### High Frequency of Recombination in *Kitasatospora* Genomes

**FIGURE 7:**
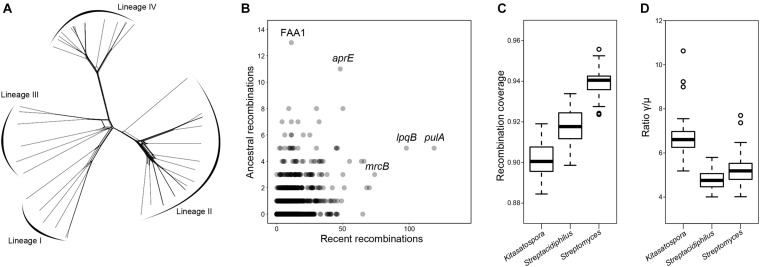
Extensive recombination in *Kitasatospora*. **(A)** Phylogenetic network inferred with the concatenation of single-copy core genes. Lines between splits show where recombination has occurred. **(B)** Core genes that have undergone recent and ancestral recombination. Horizontal and vertical axes show the estimated number of recent and ancestral recombinations, respectively. Names of some of the most frequently recombined genes are shown. **(C,D)** Comparison of the recombination coverage and the ratio γ/μ between genera, respectively. These two parameters were calculated from 50 subsets each containing 50 different species (pairwise ANI < 95%; 20 for genus *Kitasatospora*, 20 for genus *Streptomyces*, and 10 for genus *Streptacidiphilus*) randomly selected from the genome dataset. The boxplot shows the median, and the first and third quartiles as the lower and upper hinges. Outliers are indicated as dots. All values are significantly positive (*p* < 0.001, paired two-tailed Student’s *t*-test).

Recombination is known to contribute to the evolution of streptomycetes, which has been reported to frequently recombine within and between species (Doroghazi and Buckley, 2010; Cheng et al., 2016). Here, we sought to determine the frequency and extent of recombination in *Kitasatospora*. Firstly, calculation of the pairwise homoplasy index (PHI) (Bruen et al., 2006) revealed evidence for significant recombination in the core genome (1,274 single-copy core genes; *p*-value = 0.0). This could be visualized by NeighborNet network (Figure 7A), which showed a reticulate structure due to non-vertical inheritance in phylogeny (Huson and Bryant, 2006). This observation was further supported by the fastGEAR analysis (Mostowy et al., 2017), which showed that more than 98% of the core genes have been affected by recombination in the evolutionary history. In total, 1,243 (97.6%) genes were involved in recent recombination and 518 (40.7%) genes in ancestral recombination (Figure 7B). Specially, genes *pulA* (encoding a pullulanase, EC 3.2.1.41), *lpqB* (encoding a putative lipoprotein), and *mrcB* (encoding a penicillin-binding protein) have undergone 119, 98, and 74 recent recombinations, respectively. FAA1 (encoding a long-chain fatty acid-CoA ligase) and *aprE* (encoding an alkaline serine protease) have undergone 13 and 11 ancestral recombinations.

We also sought to quantify the impact of recombination on the genetic diversity of *Kitasatospora* (Figures 7C,D). The recombination coverage (c), the fraction of the genome whose diversity was derived from recombination events since the last common ancestor of the population, was estimated to be 0.90 ± 0.003. The relative rate of recombination to mutation (γ/μ) was estimated to be 6.7 ± 0.90. Interestingly, there were significant differences in these two parameters between the three genera (*p* < 0.001, paired two-tailed Student’s *t*-test): *Kitasatospora* has the lowest *c* value, but the highest γ/μ ratio.

## Discussion

While *Streptomyces* is the model of bacterial physiology and an important resource of antibiotic discovery, its close relatives are rarely studied. To the authors’ knowledge, this study represented the first systematic comparative genomic study of the *Kitasatospora* genus, a close relative of *Streptomyces*. Our phylogenomic analysis confirmed the taxonomic status of *Kitasatospora* as a separate genus distinct from *Streptomyces*. This result is consistent with the studies based on multi-locus sequence analysis (Labeda et al., 2017). Our phylogenomic analysis also revealed discrepancies between the classification and phylogenetic positions for a few strains, suggesting potential classification errors (Figures 1, 4). For example, strain DSM 106435, the type strain of *Streptacidiphilus bronchialis* (Nouioui et al., 2019), may represent a new genus (Figure 1). Furthermore, our analyses also revealed differences between the GTDB classification and the 16S rRNA gene-based taxonomy (Ezbiocloud). For example, strains DSM 44781, DSM 44780, NRRL B-11411, and NRRL S-384 were all assigned to *K. cineracea* with inner ANI > 95.8% by GTDB, but they were identified into three different species by Ezbiocloud based on 16S rRNA gene, although the gene identity >98.5%. Strains MF730-N6 and CB02891 were assigned as *K. cheerisanensis* and *K. griseola* based on 16S rRNA gene, respectively. However, the 16S rRNA gene identity between these two strains was >99.8% and the pairwise ANI value was >97.3%, suggesting these two strains should belong to the same species. Generally, phylogenomics has surpassed 16S rRNA gene-based approaches in terms of elucidating phylogenetic relationships and facilitated accurate (re)classification of microbes (Zhang et al., 2016; Hinger et al., 2020).

Our comparative genomic analyses also revealed genome content differences between genera. Interestingly, the abundance of genes of COG4934 (serine protease, subtilase family) showed different abundance in different genera: on average 10.6 copies in *Streptacidiphilus*, 4.7 copies in *Kitasatospora*, and 1.6 copies in *Streptomyces*. COG4934 was among the top ranked COGs that were correlated with acidophilic bacteria and archaea (Gonzalez and Zimmer, 2008), and has been considered as the acidity linked gene, although the molecular mechanism has not been determined. The genus *Streptomyces* is regarded to prefer neutral to alkaline environmental pH (optimal pH range: 6.5–8.0), although they commonly occur at remarkably variable pH conditions, and the genus *Streptacidiphilus* is more acidophilic with optimum growth at pH 4.5–5.5, while the preferred pH for genus *Kitasatospora* has not been determined (Goodfellow et al., 2012). Based on these, we speculated that the optimal growth pH of the genus *Kitasatospora* is between those of genera *Streptomyces* and *Streptacidiphilus*. In addition, this COG showed similar abundance in *Kitasatospora* Lineage I to in *Streptacidiphilus*. Therefore, it is possible that, similar to *Streptacidiphilus*, *Kitasatospora* Lineage I prefers acidic environments. Since *Kitasatospora* is closely related to *Streptacidiphilus* and Lineage I is the earliest diverged lineage in *Kitasatospora*, it is possible that the last common ancestor of *Kitasatospora* and *Streptacidiphilus* had abundant COG4934 and a few *Kitasatospora* lineages lost a few copies of COG4934 after their divergence from Lineage I. In addition, we have also detected a series of COGs that perhaps associated with phenotypic traits (type II fatty acid biosynthesis, carnitine metabolism, etc). We also noticed that genes of COG1545 that were largely absent in most *Kitasatospora* strains but were quite abundant in the other two genera. It has been reported that proteins of COG1545 were involved in synthesis of 2,4-Diacetylphloroglucinol, a low-molecular weight polyketide antibiotic produced by *Pseudomonas brassicacearum* strains (Mandryk-Litvinkovich et al., 2017), suggesting another obvious difference between genera. Nevertheless, more experimental evidences, both morphologically (e.g., morphological characteristics revealed by transmission electron microscopy) and biochemically, were needed to verified these differences, especially between *Kitasatospora* and *Streptomyces*.

Though there were inter-genus genome content differences, *Kitasatospora* genomes possessed high numbers of smBGCs, which were higher than those from *Streptomyces* and *Streptacidiphilus*. Since the cluster number may be affected by broken or merged clusters due to low genome assembly quality, we checked the potential correlations between the number of smBGCs and the number of contigs per genome. It was shown that the smBGC number was not correlated with the contig number (*R*^2^ = 0.078, *p* = 0.035), but rather positively correlated with the genome size (*R*^2^ = 0.42, *p* = 8.0e-07). Similarly, the total smBGC length and ratio of smBGC length were also bigger in *Kitasatospora* than the other two genera. A noticeable upward trend of these smBGCs were found during the evolutionary history, and most of the gene gain events occurred at terminal nodes, suggesting HGT events that happened recently may increase the diversity of secondary metabolism in *Kitasatospora*. Furthermore, it was also noted that most of smBGCs found in *Kitasatospora* were different from *Streptomyces* and from those reported, strongly suggesting *Kitasatospora* is a valuable and new source for antibiotic discovery in the future.

*Streptomyces* has long been considered as an interesting model for exploring patterns of microbial differentiation in terms of genetic and ecological diversity. Until now, progresses have been made in studies of *Streptomyces* evolution, revealing a high rate of horizontal gene transfer and also a widespread homologous recombination (Andam et al., 2016; McDonald and Currie, 2017; Tidjani et al., 2019). However, little is known about *Kitasatospora*. In this study, the pan-genome analyses revealed a massive gene flux in *Kitasatospora* comparable to that of *Streptomyces*. Estimation of recombination events revealed high level of recombination in *Kitasatospora* as well as in the two related genera, and the recombination coverage (c) and rate of recombination to mutation (γ/μ) are among the highest values for bacteria reported so far (Lin and Kussell, 2019; Park and Andam, 2020; Li et al., 2021).

Collectively, this study provides comprehensive comparative genomic insights into the assessment of the phylogenomic relationship and diversity of members of the genus *Kitasatospora*, and also provides evidence that supported *Kitasatospora* as a separate genus, with genomic features different from other genera in the family. This study also suggests that *Kitasatospora* is a valuable resource for the natural product discovery, which should be further experimentally confirmed in the future.

## Data Availability Statement

The datasets presented in this study can be found in online repositories. All data analyzed during this study are available through NCBI GenBank database, and are accessible through the accession numbers listed in Supplementary Table 1.

## Author Contributions

YL and B-BX contributed to conception and design of the study. YL performed the main bioinformatic analyses and wrote the draft manuscript. MW, Z-ZS, and B-BX interpreted and discussed the results and revised the manuscript. All authors contributed to manuscript revision, read, and approved the submitted version.

## Conflict of Interest

The authors declare that the research was conducted in the absence of any commercial or financial relationships that could be construed as a potential conflict of interest.

## Footnotes

1 https://www.ncbi.nlm.nih.gov/genomes

2 https://cran.r-project.org/web/packages/fpc/

3 https://github.com/vegandevs/vegan

## Supplementary Material

Supplementary Figure 1 Variations in genome size **(A)** and G + C content **(B)** between genera in family *Streptomycetaceae*.

Supplementary Figure 2 Hierarchical cluster analysis based on the presence or absence of dispensable genes.

Supplementary Figure 3 Comparison of the total number **(A)**, the total size **(B)**, and percentage of size **(C)** of smBGCs between genera.

Supplementary Figure 4 Distribution of biosynthetic gene clusters among strains of *Kitasatospora*.

Supplementary Figure 5 Ancestral reconstruction of smBGCFs in *Kitasatospora* using COUNT software.

Supplementary Figure 6 Genetic organizations of the BGCs discussed in the work.

Supplementary Table 1 Genomic features of *Kitasatospora* strains used in this study.

Supplementary Table 2 COGs contributing most significantly (>0.05%, *p* < 0.01) to differences across genera.

Supplementary Table 3 COGs contributing most significantly (>0.05%, *p* < 0.01) to differences across lineages within *Kitasatospora*.
