# Supplementary material for: Comparative Genomic Insights Into the Taxonomic Classification, Diversity, and Secondary Metabolic Potentials of Kitasatospora, a Genus Closely Related to Streptomyces
Source: Front Microbiol. 2021 Jun 14;12:683814. doi: 10.3389/fmicb.2021.683814 (PMC8236941; doi:10.3389/fmicb.2021.683814)
Supplement: Supplementary Figure 1 — Variations in genome size (A) and G + C content (B) between genera in family Streptomycetaceae. [file Presentation_1.PDF]

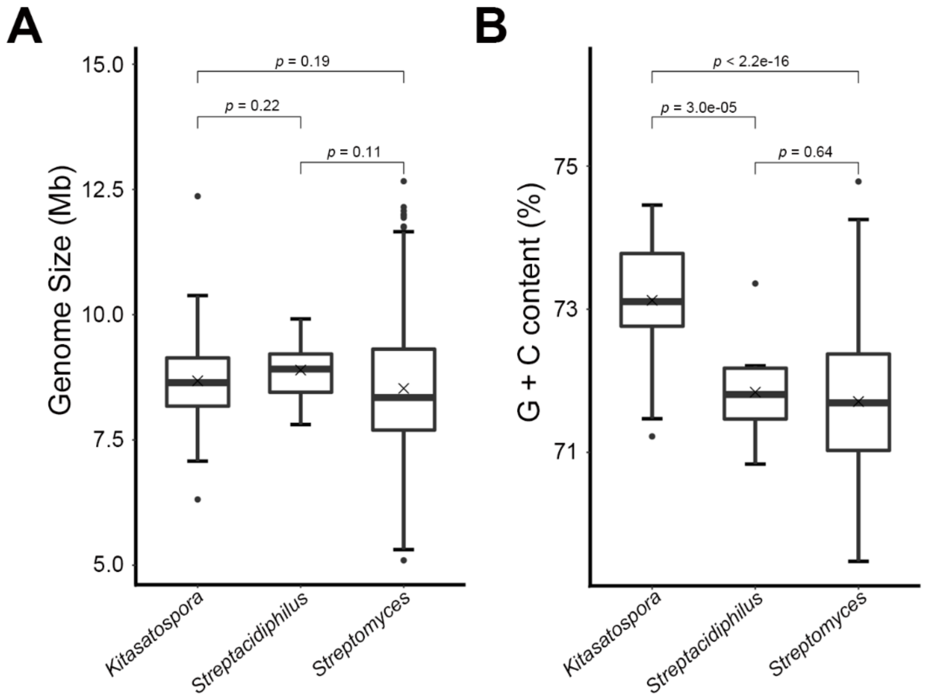

Supplementary Figure 1. Variations in genome size (A) and G+C content (B) between genera in family *Streptomycetaceae*. The boxplot shows the median, and the first and third quartiles as the lower and upper hinges. Outliers are indicated as dots, and mean values are indicated as crosses. The indicated *p* values were calculated using Wilcoxon test.

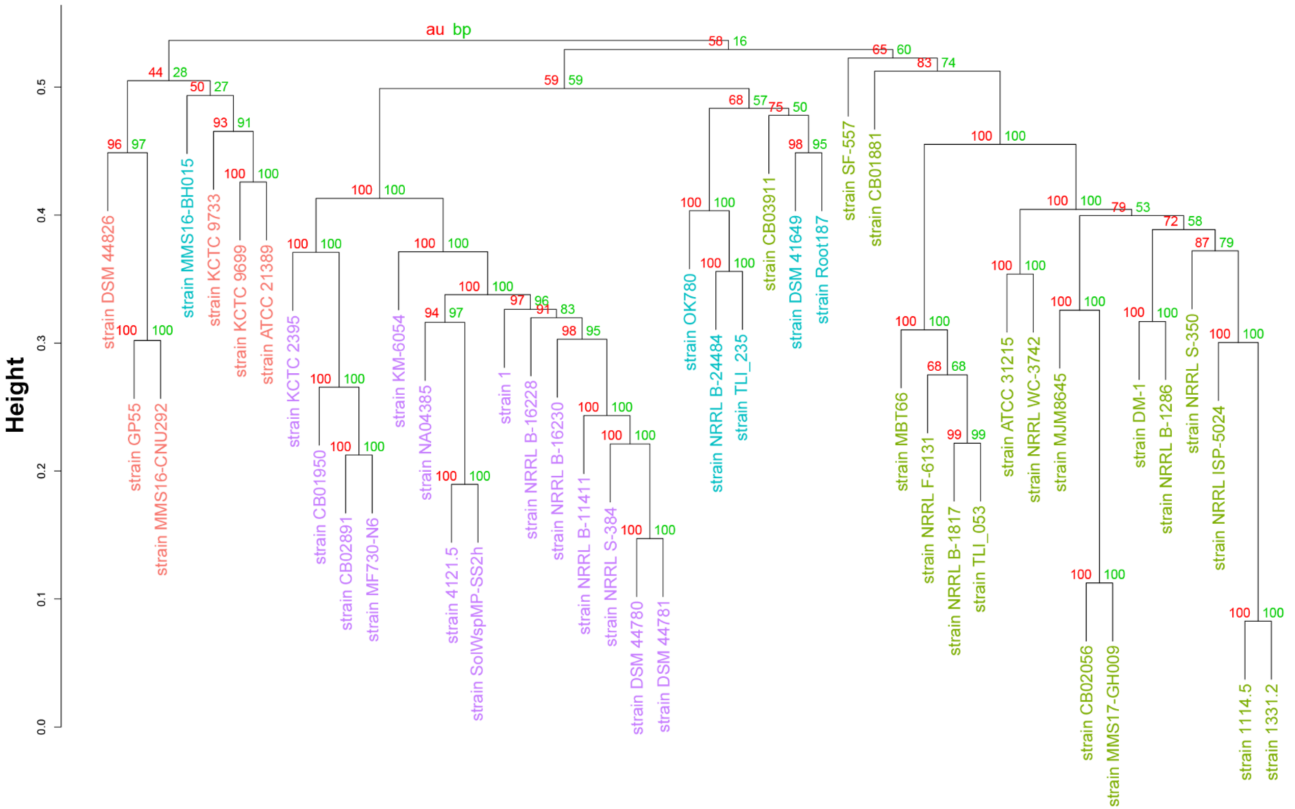

Supplementary Figure 2. Hierarchical cluster analysis based on the presence or absence of dispensable genes. Two bootstrap resampling methods from 1,000 replicates were employed to assess the uncertainty in the hierarchical cluster analysis: approximately unbiased (au) in red and bootstrap probability (bp) in green. Height indicates the dissimilarity between genomes. Four major lineages are presented by different label colors according to Figure 4.

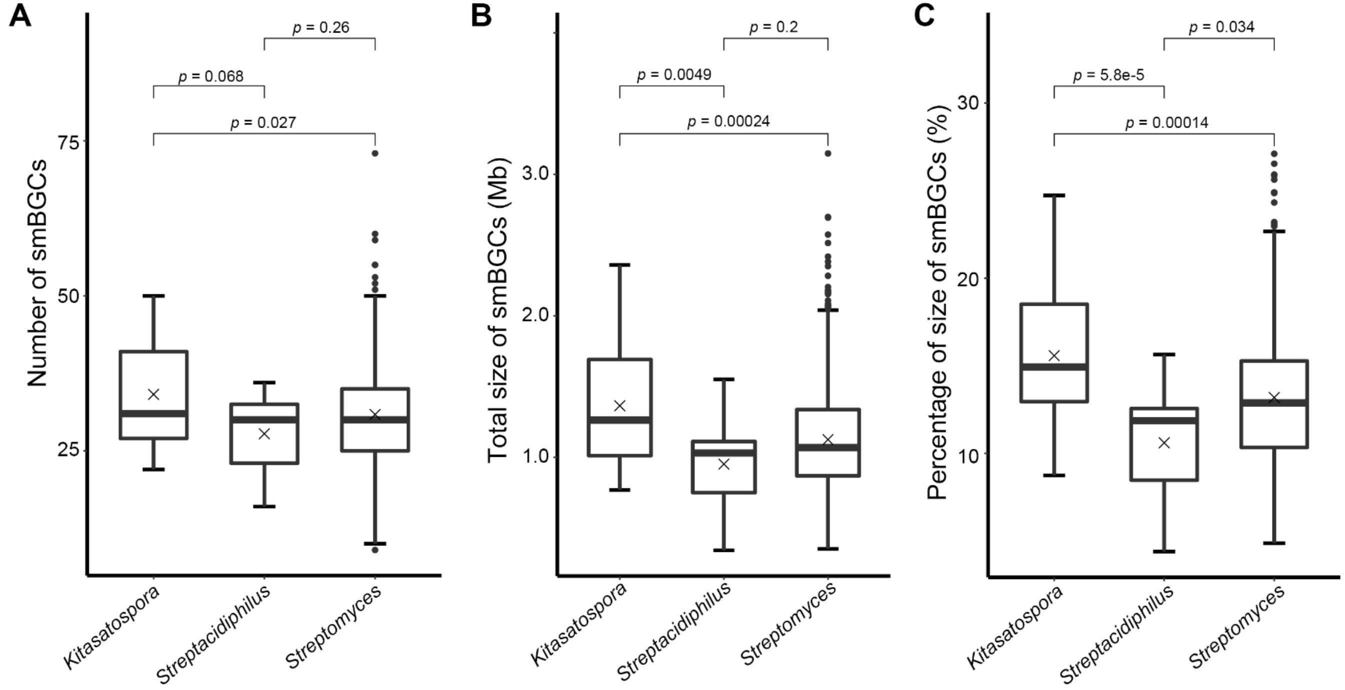

Supplementary Figure 3. Comparison of the total number (A), the total size (B), and percentage of size (C) of smBGCs between genera. The indicated *p* values were calculated using Wilcoxon test. The boxplot shows the median, and the first and third quartiles as the lower and upper hinges. Outliers are indicated as dots, and mean values are indicated as crosses.

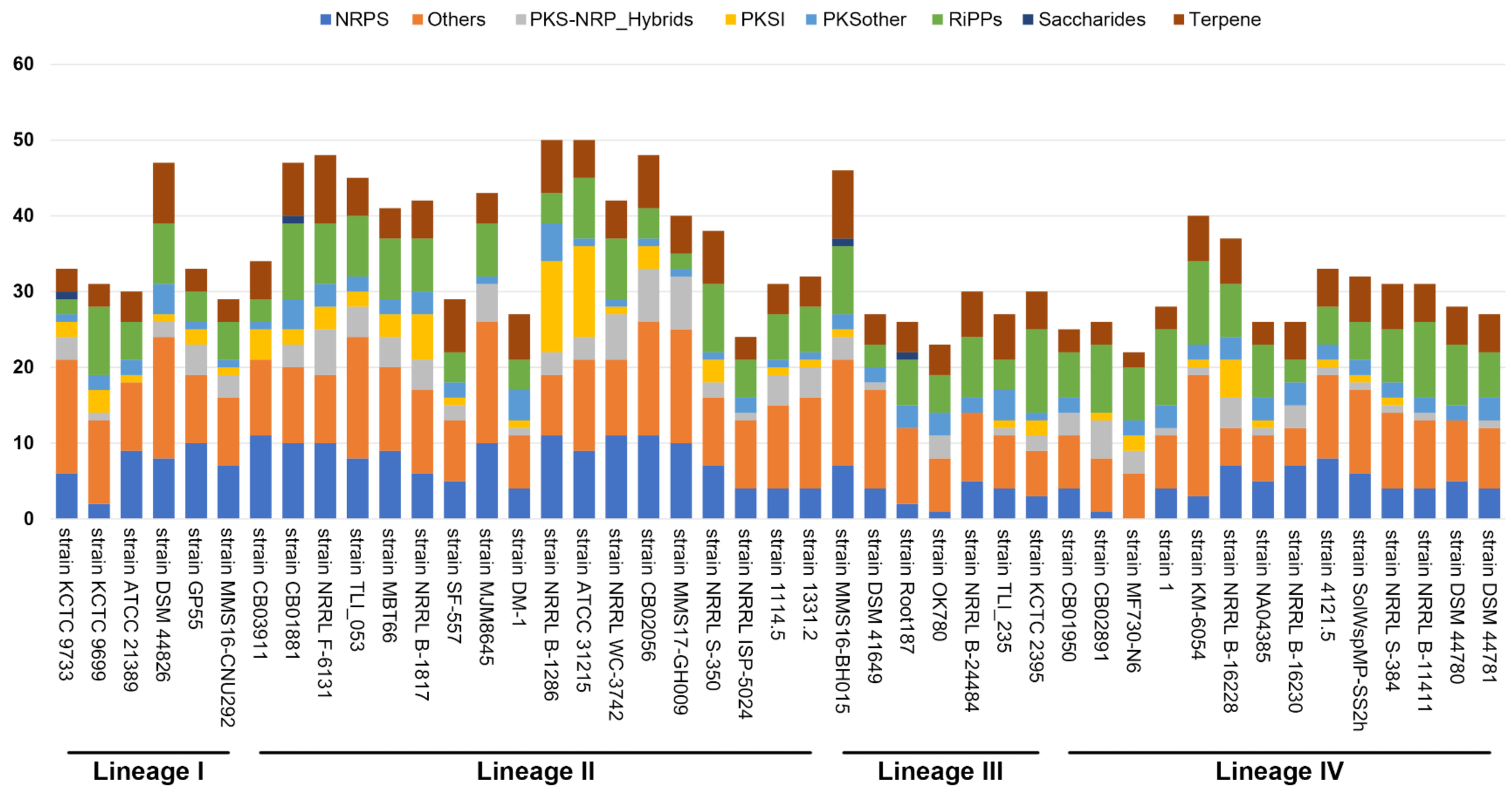

Supplementary Figure 4. Distribution of biosynthetic gene clusters among strains of *Kitasatospora*. Colors indicate the type of BGC annotated by antiSMASH.

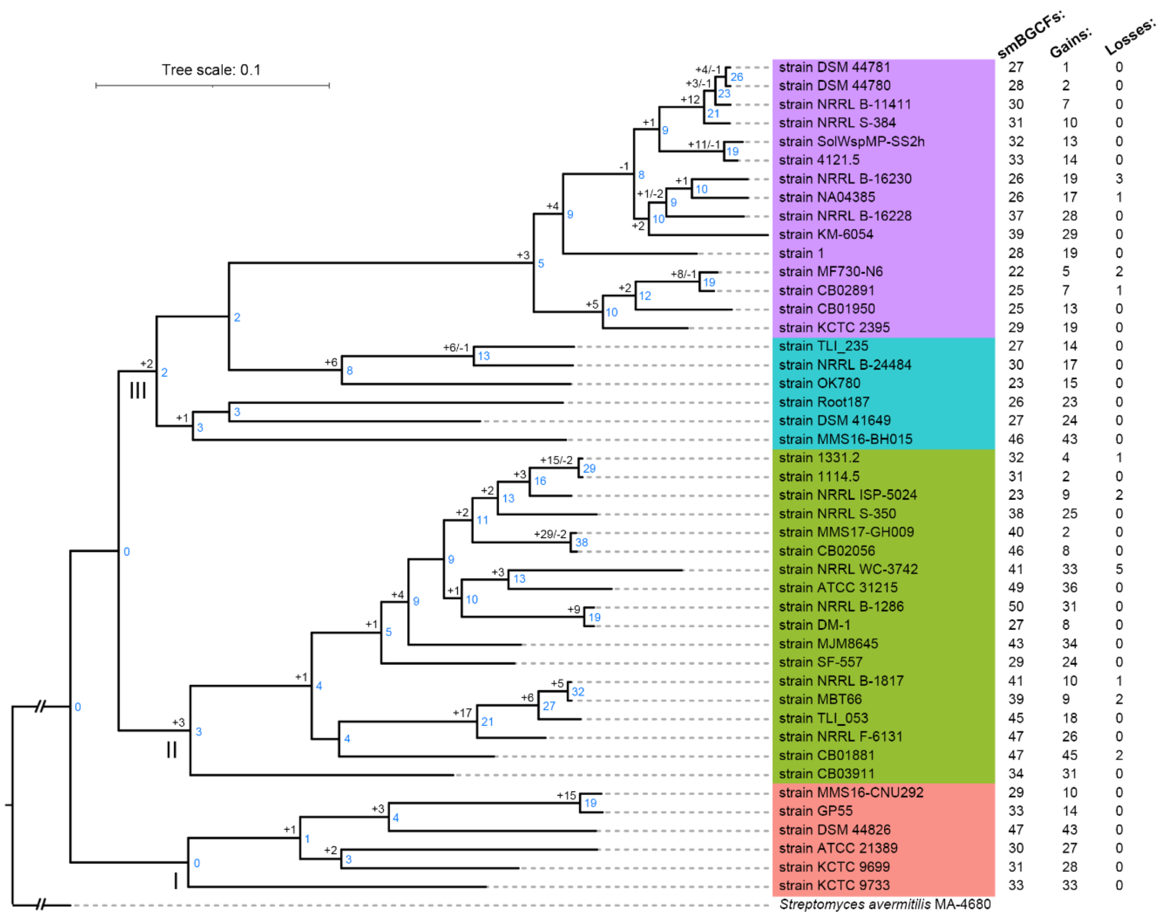

Supplementary Figure 5. Ancestral reconstruction of smBGCFs in *Kitasatospora* using COUNT software. The tree topology is derived from Figure 4. The numbers of gain and loss events are shown at each lineage of the tree. "+"s represent gain events and "–"s represent loss events. The numbers in blue represent the total numbers of smBGCFs at nodes. Four major lineages are presented by different background colors according to Figure 4, and "I", "II," or "III" indicates genetic clades. The scale bar indicates 10% sequence divergence.

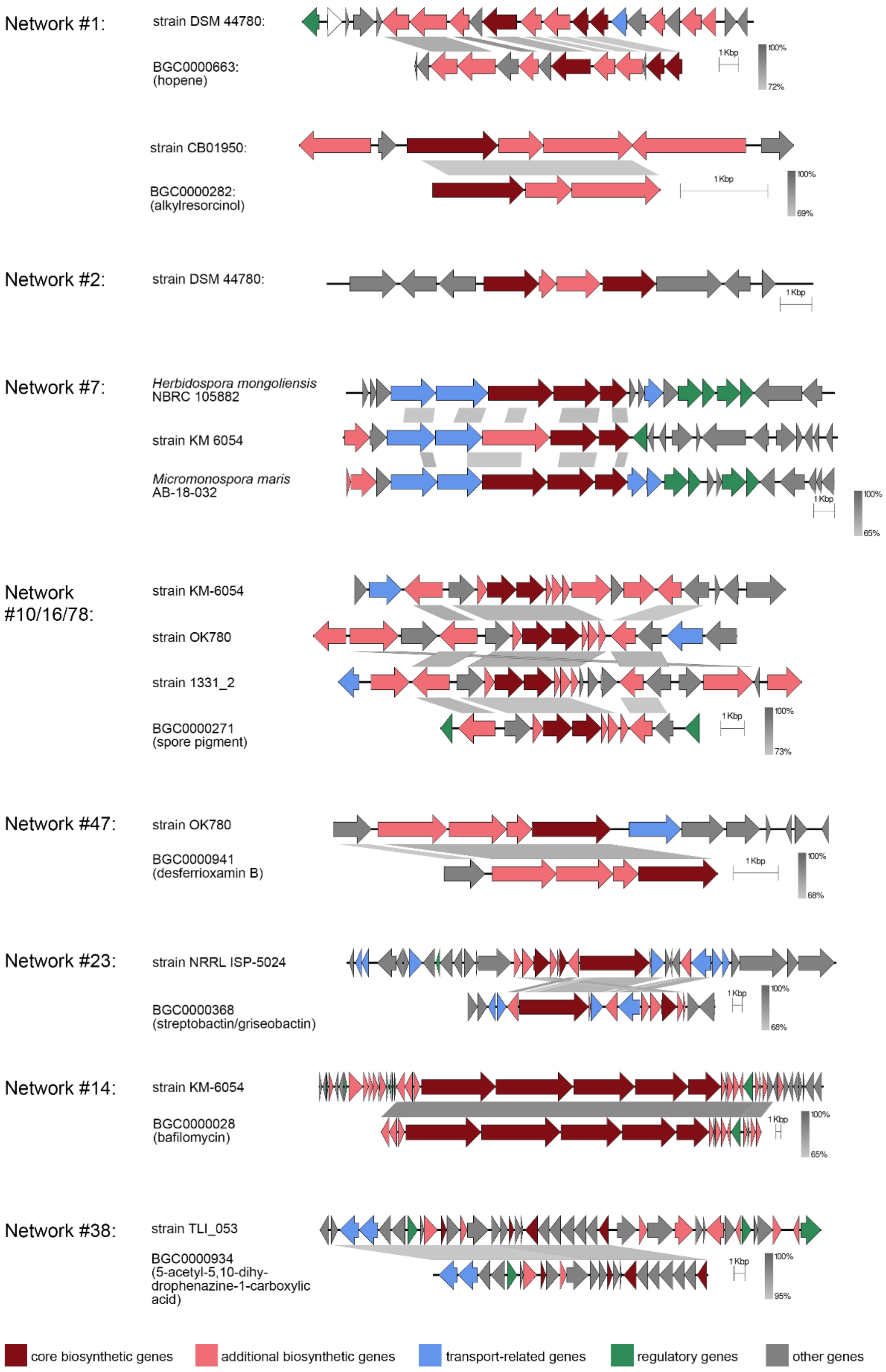

Supplementary Figure 6. Genetic organizations of the BGCs discussed in the work. The figure was obtained by EasyFig, where gray-scale bars represent regions of shared similarity according to BLASTn.
